# Monitoring Monoclonal Antibody Delivery in Oncology: The Example of Bevacizumab

**DOI:** 10.1371/journal.pone.0072021

**Published:** 2013-08-12

**Authors:** Guillaume Nugue, Marie Bidart, Marie Arlotto, Mireille Mousseau, François Berger, Laurent Pelletier

**Affiliations:** 1 Grenoble Institut des Neurosciences, Grenoble, France; 2 Centre Hospitalier Universitaire, Grenoble, France; 3 Université Joseph Fourier, Grenoble, France; Ospedale Pediatrico Bambino Gesu', Italy

## Abstract

Developing therapeutic monoclonal antibodies paves the way for new strategies in oncology using targeted therapy which should improve specificity. However, due to a lack of biomarkers, a personalized therapy scheme cannot always be applied with monoclonal antibodies. As a consequence, the efficacy or side effects associated with this type of treatment often appear to be sporadic. Bevacizumab is a therapeutic monoclonal antibody targeting Vascular Endothelial Growth Factor (VEGF). It is used to limit tumor vascularization. No prognosis or response biomarker is associated with this antibody, we therefore assessed whether the administration protocol could be a possible cause of heterogeneous responses (or variable efficacy). To do this, we developed a bevacizumab assay with a broad sensitivity range to measure blood bevacizumab concentrations. We then analyzed bevacizumab concentrations in 17 patients throughout the first quarter of treatment. In line with previously published data, average blood concentrations were 88+/−27 mg/L following the first dose administered, and 213+/−105 mg/L after the last (6^th^) dose administered. However, the individual values were scattered, with a mean 4-fold difference between the lowest and the highest concentration for each dose administered. We demonstrated that the bevacizumab administration schedule results in a high inter-individual variability in terms of blood concentrations. Comparison of assay data with clinical data indicates that blood concentrations above the median are associated with side effects, whereas values below the median favor inefficacy. In conclusion, bevacizumab-based therapy could benefit from a personalized administration schedule including follow-up and adjustment of circulating bevacizumab concentrations.

## Introduction

Over the last decade, significant advances have been made in the care of cancer patients. Oncology's therapeutic arsenal has been expanded through the emergence of monoclonal antibodies (mAbs). mAbs are produced by a single B cell immortalized by fusion with a myeloma cell, in a process developed by Kohler in 1975 [1]. In contrast to chemotherapy, which has remarkable effects but significant toxicity, mAbs have the advantage of being highly specific. They thus represent a targeted therapy, and are likely to have limited toxicity and be applicable as part of a personalized medicine strategy.

The Vascular Endothelial Growth Factor (VEGF) pathway was recognized as a key regulator of angiogenesis in. This led to the development of several VEGF-targeting agents, including bevacizumab (Avastin®, Roche-Genentech) [3]. Bevacizumab is a humanized anti-VEGF monoclonal antibody, which neutralizes VEGF. It thus inhibits angiogenesis and limits tumor growth [2], [3]. Bevacizumab was the first anti-angiogenesis agent approved by the Food and Drug Administration (FDA) for the treatment of metastatic colorectal cancer in 2004 [4]. Since then, bevacizumab has received additional FDA approval, including for glioblastoma in 2009 [5].

Despite an overall benefit of bevacizumab in the treatment of different tumors, clinical outcomes can be highly variable, with some patients responding remarkably well to anti-angiogenic therapy, while others do not [6], [7]. Furthermore, side-effects such as vascular disorders (bleeding, phlebitis, embolism) often lead to definitive cessation of the treatment [8].

Because of this heterogeneous response, the real clinical impact of bevacizumab remains unclear. For example, although bevacizumab delays the progression of brain and breast cancers, it does not improve overall survival [9], [10].

One way of assessing the clinical efficacy of a drug is to characterize its pharmacokinetics (PK). The antibody concentration and the kinetics of monoclonal antibody elimination are modulated by several parameters (body weight, gender, liver function) [11], [12]. Thus, modulation of the PK of bevacizumab could explain the inter-individual variability observed in patients. Roche-Genentech have reported a mean half-life of bevacizumab close to 20 days [13], however, large individual differences were noted, with a half-life ranging between 11 and 50 days. In spite of this, a standardized administration protocol is recommended [13]. Presumably, inter-individual variability in bevacizumab PK could cause variable responses to the treatment. When treatment is administered every other week, a patient for whom the half-life is 50 days would present an excess of circulating antibody from the second dose. This could be the reason for the vascular disorders noted as side effects during therapy. In contrast, a patient for whom the half-life is only 11 days will rapidly clear bevacizumab, and this could impede treatment efficacy. Since the dawn of the therapeutic mAb era, personalized treatment regimes have not been applied, despite significant PK variability. Rapid assays must therefore be developed to monitor concentrations in patients' blood during treatment. This will allow the dose of the drug to be adapted to the patient's own pharmacokinetic profile. This is essential if a real therapeutic effect is to be observed. A first step in this process requires precise monitoring of bevacizumab concentrations throughout treatment. In this article, we describe an assay based on suspension array technology [14]. The assay we have developed is sensitive, rapid, accurate and adapted to clinical use. The approach was validated by evaluating bevacizumab blood levels in a cohort of brain- and breast-cancer patients treated with bevacizumab.

## Materials and Methods

### Patients

Patients included in this study were men and women between 39 and 79 years old, treated with bevacizumab for brain- or breast-cancer (n = 17). For the pharmacodynamic study, patients with breast cancer were excluded (n = 3) and clinical data was confidential for one brain tumor patient. Analysis was therefore performed on 13 glioma patients. All patients provided written informed consent for participation in this study (CBR.GSI.ENR.003 V4, Grenoble University Hospital). The informed consent document and the study as a whole were approved by the hospital ethics committee (COMITE DE PROTECTION DES PERSONNES).

### Treatment and Samples

Bevacizumab was intravenously infused at 10 mg/kg of body weight every two weeks. Blood samples were taken just before bevacizumab infusion.

### Bead Coupling

Recombinant human VEGF (2–10 µg; PHC9393; Invitrogen) was conjugated to Bio-Plex COOH Pro Magnetic beads (Bio-Rad). Briefly, beads were washed once in washing buffer (PBS, 0.05% tween-20, pH 7.4). Beads were incubated under slow rotation with activation buffer (0.1 M NaH_2_PO_4_, pH 6.2), 5 mg/ml S-NHS (P24510; Thermo Scientific) and 5 mg/ml EDAC (P77149; Thermo Scientific) for 20 min at room temperature (RT), and washed twice in PBS. Beads were then incubated for 2 hours with rhVEGF in PBS, with mixing by rotation at RT. Beads were washed once in washing buffer and incubated for 30 mins in blocking buffer (PBS, 1% BSA, 0.05% Sodium Azide, pH 7.4). Beads were stored at 4°C in storage buffer (PBS, 1% BSA, 0.05% Sodium Azide, pH 7.4).

### Bevacizumab Assay Procedure

Bevacizumab was assayed by suspension array technology. Briefly, 5,000 VEGF-beads were deposited per well in Bio-Plex pro flat-bottom plates (Bio-Rad). Beads were washed twice with 75 µl of wash buffer (PBS-0.05%Tween-20, pH 7.4) on a wash station (Bio-Plex Pro II; Bio-Rad). Beads were then incubated with appropriately PBS-diluted serum for 30 min under slow agitation at RT. Beads were then washed three times on a wash station and incubated with phycoerythrin-conjugated (PE) anti-human IgG (Sigma-Aldrich) for 30 min under slow agitation at RT. Beads were once again washed three times on a wash station. PE binding was measured on a Bio-Plex 220 (Bio-Rad) and analyzed using Bio-Plex Manager 5.0 software (Bio-Rad). Bevacizumab used as standard was purchased from Roche-Genentech, and stored at 4°C.

### Assay Validation

Assay characterization and validation was performed according to the guidelines for method validation in medical biology [15]. Briefly, the lowest limit of quantification (LLOQ) was calculated as the mean signal background plus three standard deviations. The highest limit of quantification (HLOQ) was calculated as the mean maximum fluorescence minus three standard deviations. The coefficient of variation (CV) was calculated for each concentration point by dividing the mean by the standard deviation. The inaccuracy (or bias) was calculated for each concentration point as the mean minus the value divided by the value.

### Statistical Analysis

Graphpad Prism software (version 4)was used for statistical analysis. The bevacizumab concentration parameter was compared for each patient sample using the Kruskal-Wallis test. Statistical significance was defined as *p*<0.05.

## Results

### Developing and Validating the Bevacizumab Assay

The first step of our work was to develop a bevacizumab assay using a suspension array. This required covalent linkage of rhVEGF to beads which could then be used to trap bevacizumab in samples. Bevacizumab binding was quantified using a PE-conjugated secondary antibody directed against human IgG.

In line with the instructions for the Bio-Plex amine coupling kit (Bio-Rad), we adapted several parameters (number of beads per well, amount of rhVEGF coupled to beads, secondary antibody concentration) to optimize the assay. The maximum of this range corresponds to twice the maximum expected bevacizumab concentration in serum. For our assay, we chose to coat beads with 10 µg rhVEGF (see material and methods section), to use 5,000 beads per well and 1,000-fold diluted sample.

**Figure 1:**
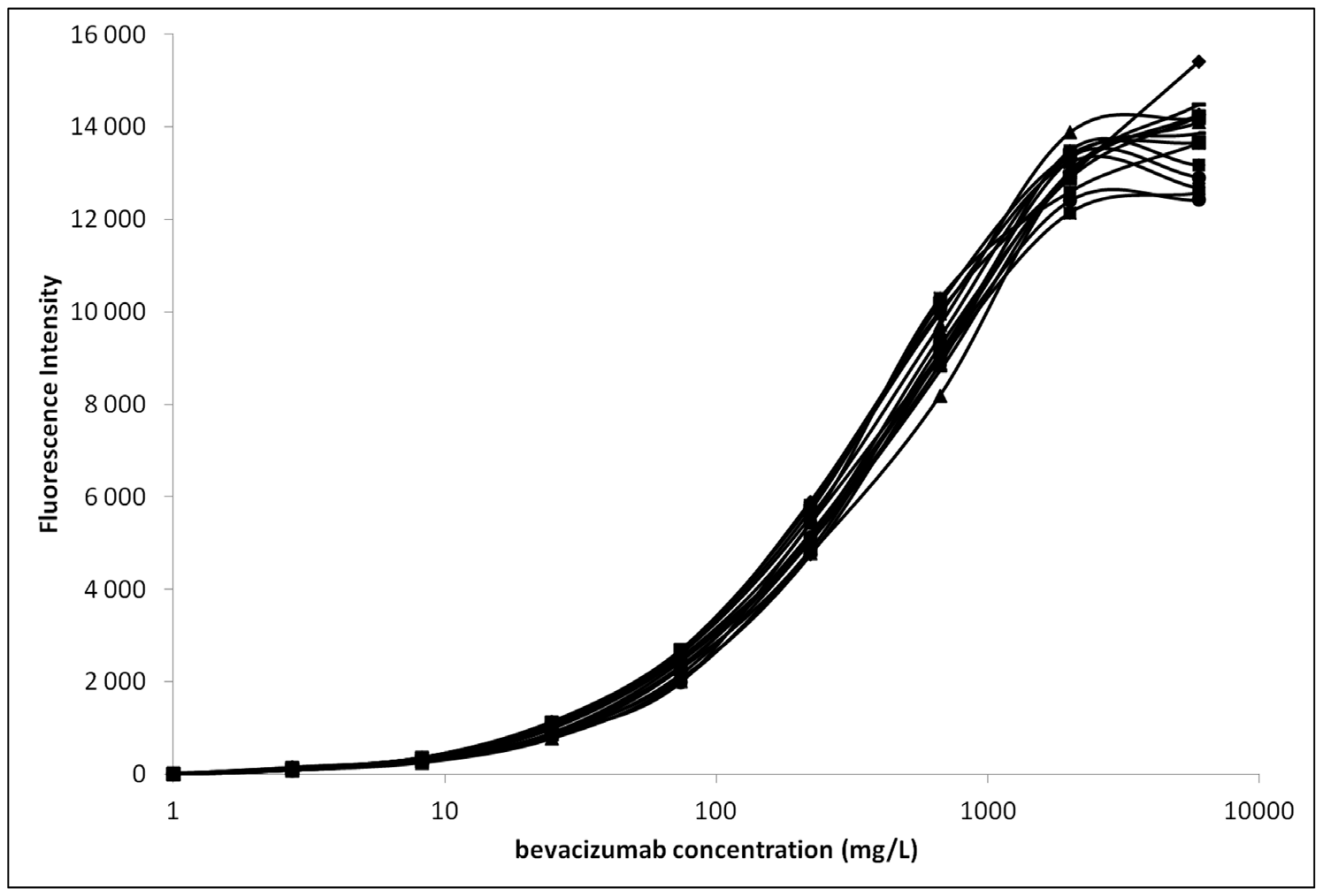
Bevacizumab assay calibration. Fluorescence intensity was measured on 15 independent assays over a large bevacizumab range (0–6 mg/mL).

Next, we qualified the assay by defining LHOQ, LLOQ, CV and inaccuracy (or bias) (Table 1). These parameters were defined on a series of 15 independent assays (figure 1). The CV varied from 6 to 25% over the 0.001 mg/L to 6 mg/L range and remained below 15% from 0.008 to 6 mg/L. According to the guidelines for biomedical assays, the assay is valid only with a CV below 15%. Our assay is therefore valid from 0.008 to 6 mg/L. The LLOQ and LHOQ were determined as 0.008 and 1 mg/L, respectively, and the bias defining accuracy was 1.48% (+/−1.83).

**Table 1:** Validation of bevacizumab assay (n = 15).

bevacizumab (mg/L)	CV (%)	Bias (%)
6	**6.03**	NA
2	**6.43**	4.51
0.7	**6.86**	3.11
0.2	**6.99**	0.04
0.07	**10.03**	−0.6
0.025	**12.87**	2.25
0.008	**12.15**	0.01
0.003	20.28	0.47
0	24.92	NA

The coefficient of variation (CV) was calculated for each concentration point by dividing the mean by the standard deviation. The assay was validated for CVs under 20% (bold). Inaccuracy (bias) was calculated (mean minus value divided by value) only over the validated range.

NA: not applicable.

### Variable Bevacizumab Pharmacokinetics in Patients

**Figure 2:**
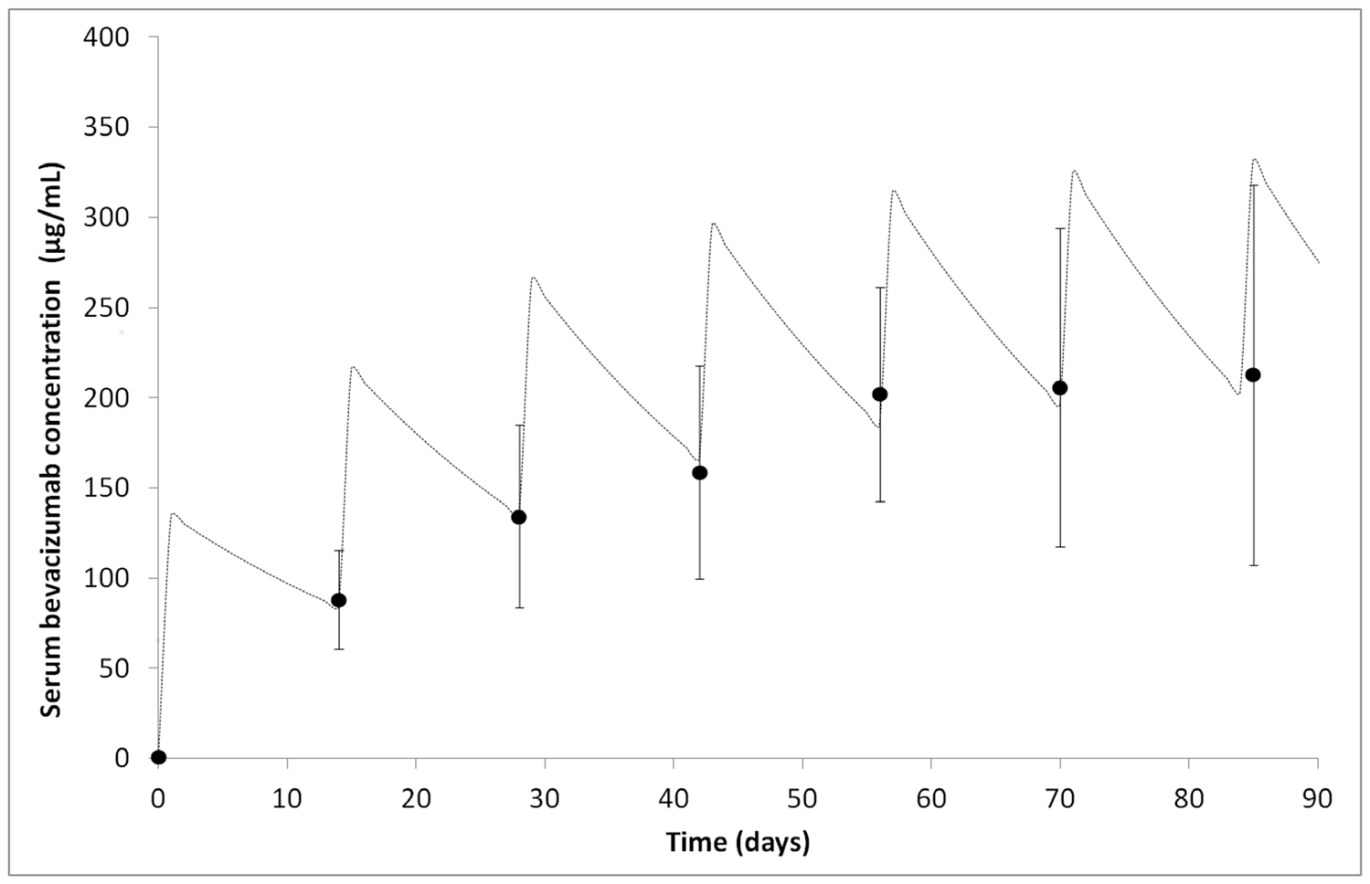
Evolution of blood bevacizumab concentrations over the first 3 months of treatment. Points represent the mean bevacizumab concentrations for the 17 patients (+/− SD).The dotted line corresponds to the theoretical bevacizumab concentration in a 70-kg patient, with treatment by 7 repeat administrations of 10 mg/kg every other week. The theoretical value was calculated using the bevacizumab half-life reported by Roche-Genentech (20 days).

Once we had developed and validated our assay, we measured bevacizumab concentrations in patient sera. Seventeen patients were analyzed over the first three months of bevacizumab treatment (6 complete treatment cycles; Table 2). Blood samples were taken just before bevacizumab administration every second week. Before treatment was initiated, no bevacizumab was detected in serum. As expected, following repeated administration, the serum concentration reached a steady state. At the sixth dose, the concentration was precisely 95% of the plateau value (figure 2). If the half-life for bevacizumab is 20 days (Roche-Genentech data) and 10 mg/kg is administered at each dose, the plateau for bevacizumab concentration should shift from 203 mg/mL to 330 mg/L, just before and just after administration, respectively. The concentration measured at the plateau just prior to administration was 213+/−105 mg/L, which is very close to the theoretical value.

**Table 2:** Patient characteristics.

Patient	Age	Sex	Weight	Disease	Treatment	Bevacizumab at70 days (mg/L)
1	78	F	–	Breast cancer	Paclitaxel-bevacizumab	93
2	60	M	64	Glioma	Bevacizumab	380*
3	49	F	57	Glioma	Irinotecan-bevacizumab	252
4	58	M	75	Glioma	Irinotecan-bevacizumab	205
5	56	M	69	Glioma	Irinotecan-bevacizumab	229*
6	52	M	79	Glioma	Irinotecan-bevacizumab	166
7	41	M	–	Glioma	Irinotecan-bevacizumab	283*
8	50	M	74	Glioma	Irinotecan-bevacizumab	121*
9	61	M	71	Glioma	Bevacizumab	313
10	56	F	102	Breast cancer	Paclitaxel-bevacizumab	131
11	59	M	63	Glioma	Irinotecan-bevacizumab	213
12	58	M	–	Glioma	Irinotecan-bevacizumab	289
13	53	M	79	Glioma	Irinotecan-bevacizumab	350
14	51	F	72	Breast cancer	Paclitaxel-bevacizumab	348
15	58	M	86	Glioma	Irinotecan-bevacizumab	355
16	56	F	82	Glioma	Irinotecan-bevacizumab	175
17	64	M	82	Glioma	Irinotecan-bevacizumab	267

Serum bevacizumab concentrations were determined at the plateau (70 days; values labeled with an asterisk correspond to 56 days). M: Male; F: Female.

When we consider individual data rather than the mean concentration for the population, we observed extensive dispersion. For example, concentrations were between 54 and 149 (88+/−27) mg/L and 73 and 411 (213+/−105) mg/L for the first and the last doses, respectively. Thus, the ratio between the lowest and highest value was 2.8 and 5.6, respectively. This is representative of all the results, which have an overall ratio of 4.3.

### Identifying Serum Bevacizumab Levels as a Pharmacodynamic Marker

**Figure 3:**
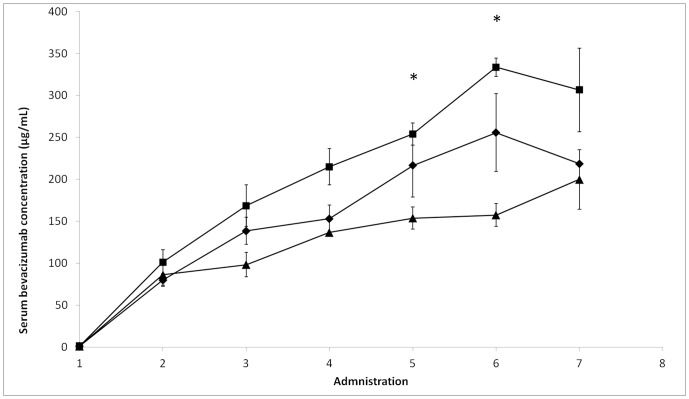
Serum bevacizumab levels as a function of treatment efficacy. (▪) mean value for patients with side effects (n = 5); (▴)mean value for patients with residual angiogenesis (n = 4); (♦) mean value for patients responding favorably to treatment (n = 4).

Patients were then classed in three groups according to clinical data: patients with side effects, non-responders and good responders. The side effects group (n = 5) was defined as patients with vascular complications, in particular epistaxis, gastrointestinal hemorrhage and high blood pressure. One case of phlebitis was also associated with the highest serum bevacizumab level (355 mg/L). The non-responder group (n = 4) presented residual angiogenesis, as assessed by MRI after three months of treatment. Patients who did not have any residual angiogenesis nor side effects were defined as good responders (n = 4). Remarkably, low serum bevacizumab concentrations were associated with a lack of efficacy, while high concentrations were associated with side effects (figure 3). Comparison of mean variances confirmed that the mean concentrations were significantly different in the three clinical groups (*p*<0.05). When the mean concentrations were examined for each dose, the groups appeared significantly different for doses 5 (*p*<0.05) and 6 (*p*<0.01). For both these doses, the side effects group was associated with the highest concentrations (254+/−13 mg/L and 333+/−11 mg/L, respectively); the non-responder group had the lowest concentrations (153+/−13 mg/L and 200+/−35 mg/L, respectively); and the responder group was associated with intermediate concentrations (216+/−37 mg/L and 218+/−0 mg/L, respectively). Thus, serum bevacizumab concentration appears to be a useful clinical pharmacodynamic marker.

## Discussion

Whatever the biological status of patients, mAb therapies are nearly always applied using a standardized administration protocol, without personalization. This study aimed to determine whether adapting bevacizumab administration only on the basis of the patient’s physical parameters (weight) could lead to treatment discrepancies.

To do this, we first developed a bevacizumab immunoassay using suspension array technology for rapid, sensitive measurement of this mAb in patient's serum. Our bevacizumab assay can be completed in 2 hours, which is a significant improvement over the 6 hours required for a similar ELISA [7]. The ELISA is accurate and reproducible between 5 and 75 mg/L [7]. Our test is also accurate and reproducible, but over a more clinically relevant quantification range (0.008 to 6 mg/L) with a CV below 15%. Our multiplex bevacizumab assay is therefore readily adaptable to clinical practice. Moreover, in contrast with ELISA, our assay can be used for multiplexed assays [14]. For example, it would be highly relevant to simultaneously measure bevacizumab and its circulating target, VEGF.

This assay was then used to monitor bevacizumab PK in a cohort of 17 glioma or breast cancer patients treated with bevacizumab. The mean concentration for our cohort was close to the previously reported theoretical concentration with intravenous infusion every two weeks at 10 mg/kg body weight (figure 2). However, we observed a wide range of serum bevacizumab concentrations with the same administration regime. For example, the concentrations ranged from 54 to 149 mg/L before the second dose, and from 73 to 411 mg/L before the final (sixth) dose was administered. These results emphasize that patients treated by applying the same protocol (10 mg/kg of body weight) do not benefit from the same efficacy of treatment.

Limited PK and pharmacodynamics data has been published on bevacizumab, and most of the information available is supplied by Roche-Genentech [13]. Moreover, inter-individual PK variability is generally not taken into account since PK is measured as the mean for a population, and is then used to determine which dose should be given to all individuals. We must now reevaluate this parameter for bevacizumab therapy. The PK parameters for bevacizumab are similar to those for IgG, for which body weight is the covariate with the greatest influence on interpatient variance [16]. This supports a treatment schedule design based on body weight. However, gender, which is associated with a difference in muscular mass; tumor burden, which correlates with a faster bevacizumab clearance [17]; or serum glutamic oxaloacetic transaminase (SGOT), indicative of impaired liver function, could also be assessed as covariates. In the Roche-Genentech study, slight differences in clearance were noted as a function of these factors [13]. However, other factors should also be studied. For example, recycling of the neonatal Fc receptor (FcRn) is a common rescue pathway for monoclonal antibodies that leads to a long half-life [18]. In 2008, Lu et al. [6] described a mathematical model to address the variability of bevacizumab PK based on five parameters (body weight, gender, albumin, alkaline phosphatase, and SGOT). They found weight and gender to be the covariates with the greatest influence on bevacizumab PK [6]. Nonetheless, these mathematical models have limits as they cannot take all possible covariates into account. For example, serum VEGF concentrations could affect bevacizumab clearance. To check this, we measured serum VEGF levels, but no correlation with PK was found (data not shown). In addition, mathematical models require numerous parameters, which are not always easy to provide in a clinical context. For simple optimization of bevacizumab scheduling and to eliminate covariation, we suggest using our bevacizumab immunoassay as a global biomarker of PK.

Beyond the description of inter-individual heterogeneity, our results raise the issue of how PK correlates with clinical outcome. This study thus questions the relevance of an administration protocol for a monoclonal antibody not associated with a companion test to monitor its PK for each patient as part of a personalized therapeutic approach. Our preliminary data strongly suggest that low bevacizumab blood levels (less than 200 mg/L just prior the 6^th^ administration) - associated with residual angiogenesis - could compromise treatment efficacy, whereas a high level (more than 250 mg/L just prior the 6^th^ administration) - close to the toxic dose - could increase the occurrence of side effects (hemorrhage, phlebitis) (Figure 3). We observed that the maximum treatment efficacy/side effects ratio appeared with a bevacizumab concentration between 200 and 250 mg/L. This therapeutic index has to be more precisely determined using a reinforced cohort, Such a deepened study would also refine the pharmacodynamics results, allowing to discriminate patients groups earlier during the treatment. Thus, the serum bevacizumab level is a candidate pharmacodynamic marker for this therapy, and should be further validated to allow tailored treatment schedules to be developed. In contrast with Lu et al. [6], our data revealed no link between tumor burden and serum clearance. Thus, further studies are required, for example, with blood samples from clinical trial patients.

In line with the well-known relationship between PK parameters and efficacy, our results, and others [8], suggest that bevacizumab concentrations could be a prognostic/response biomarker. Using PK measurements as a companion test for personalizing mAb administration could thus eliminate the current treatment bias. Patients with low levels of circulating bevacizumab could be given an increased drug dose on subsequent administration, while patients with high levels could receive less. The procedure for defining the correction factor to be applied to the doses administered should be determined in future studies using mathematical models, and with data from a larger patient cohort.

There is a huge need for prognostic biomarkers or response indicators when treating patients with bevacizumab. Currently, progression-free survival at 6 months is between 36% and 46% for bevacizumab-treated patients with glioblastoma [9], [11], but no benefit in terms of overall survival is observed. Oncologists are thus faced with a situation where they do not know the correct optimum dose for patients. Because of this, mAbs are currently prescribed as a function of maximal therapeutic dose, similar to chemotherapy. Our results suggest that patients could benefit from an early test to adapt the dose, which could improve therapeutic efficacy while decreasing side effects. The clinical endpoints for trials to define doses should move to more functional endpoints, for example considering efficacy. This would shift the focus from a maximum therapeutic dose to an optimal biological dose [19].

In conclusion, the fundamental role of bevacizumab in the therapeutic arsenal in oncology, the highly variable patient responses, and the relative lack of data on the PK for this monoclonal antibody suggest that measuring blood bevacizumab levels may be clinically relevant. We have developed a robust and sensitive bevacizumab assay which is rapid and could be suitable for use in clinical routine as part of personalized treatment.
